# Mechanical Characteristics of SPG-178 Hydrogels: Optimizing Viscoelastic Properties through Microrheology and Response Surface Methodology

**DOI:** 10.29252/ibj.24.2.110

**Published:** 2019-11-03

**Authors:** Mansooreh-Sadat Seyedkarimi, Hamid Mirzadeh, Aliasghar Mohammadi, Shadab Bagheri-Khoulenjani

**Affiliations:** 1 Polymer and Color Engineering Department, Amirkabir University of Technology, Tehran, Iran;; 2 Malek-Ashtar University of Technology, Tehran, Iran;; 3 Department of Chemical and Petroleum Engineering, Sharif University of Technology, Tehran, Iran

**Keywords:** Hydrogels, Regenerative medicine, Rheology, Tissue engineering, SPG-178

## Abstract

**Background:**

SApeptides have growing applications in tissue engineering and regenerative medicine. The application of SApeptide-based hydrogels depends strongly on their viscoelastic properties. Optimizing the properties is of importance in tuning the characteristics of the hydrogels for a variety of applications.

**Methods:**

In this study, we employed statistical modeling, conducted with the RSM and particle tracking microrheology, to investigate the effects of self-assembling SPG-178 peptide and added NaCl salt concentrations and milieu type (DI water or blood serum) on the viscoelastic properties of SPG-178 hydrogels. A central composite RSM model was employed for finding the optimum value of the parameters to achieve the highest storage modulus and the lowest tan δ.

**Results:**

Viscoelastic properties of each sample, including storage modulus, loss modulus, and tan δ, were determined. Storage modulus and tan δ were modeled, accounting for the impact of the SPG-178 peptide and NaCl concentrations and milieu type on the viscoelastic properties. It was found that the SPG-178 hydrogel storage modulus was positively influenced by the SPG-178 peptide concentration and the serum.

**Conclusion:**

A combination of microrheology and RSM is a useful test method for statistical modeling and analysis of rheological behavior of solid-like gels, which could be applied in various biomedical applications such as hemostasis.

## INTRODUCTION

Self-assembling peptides are very promising biopolymers for biomedical and tissue engineering applications^[^^1^^-^^3^^]^. SApeptides lead to the formation of peptide nanofibers, which aggregate into hydrogel scaffold networks in physiological conditions^[^^4^^-^^6^^]^.

There are various SApeptides reported in the literature. EAK16 is one of the first peptides introduced as a SApeptides. EAK16 contains self-complementary units that can spontaneously self-assemble in proper media^[^^7^^]^. RADA16 is the most investigated SApeptides and is well known for its application in hemostasis and tissue regeneration^[^^8^^-^^10^^]^. SPG-178 is the most recently introduced SApeptides with the amino acid sequence of [CH_3_CONH]-RLDLRLALRLDLR-[CONH_2_] (R, L, D, and A indicate arginine, leucine, aspartic acid, and alanine, respectively), forming a fibrillar hydrogel structure via the self-assembling process, similar to RADA16^[^^11^^]^. SPG-178 hydrogel has potential applications in tissue engineering, including vitreous substitute for eyes^[^^12^^]^, bone regeneration^[^^13^^,^^14^^]^, neurite outgrowth of the spinal motor neurons^[^^15^^]^, and rapid hemostasis^[^^16^^]^. Due to its neutral pH value in water as well as its resistance to autoclaving conditions, SPG-178 is easier to handle in comparison to RADA16-I. Besides, the stiffness of SPG-178 hydrogel is slightly higher than that of RADA16 hydrogel in similar conditions^[^^16^^,^^17^^]^.

In a variety of applications, mechanical properties of SApeptides have to be within a specified range^[^^18^^]^. Particularly for hemostasis, it is necessary to form an elastic hydrogel to prevent hemorrhage^[^^19^^]^. Therefore, it is of importance to develop a method for tailoring the mechanical properties of the SApeptides. This goal can be achieved via either functionalization and insertion of motifs into the peptide structure^[^^20^^-^^22^^]^ or adjustment of formulation and self-assembling conditions of the SApeptides, for instance, through controlling the added salt^[^^23^^-^^25^^]^ or peptide concentrations^[^^26^^,^^27^^]^.

Precise measurement of the viscoelastic properties of SApeptides is crucial for their optimization in different applications. Traditionally, viscoelastic properties are provided through rheological measurements, undertaken using rheometers. However, the traditional rheometers are limited to low frequencies. Even though low frequency range is appropriate for solid- or liquid-like behavior, there are many complex fluids with microstructural relaxations that occur at higher frequencies. On the other hand, these devices require milliliter-scale material samples, but most biological samples are available in much lower quantities. Since the stress or strain is applied from boundaries, the bulk viscoelastic properties are measured, and the heterogeneities are not probed. This is a serious limitation for non-homogeneous materials with various microstructural length scales. Moreover, the traditional rheometers are not applicable to non-conventional geometries, such as thin films or the interior of biological cells and membranes. Therefore, such limitations have motivated the development of a number of techniques to probe the viscoelastic properties of materials on microscopic length scales. These microrheological techniques can probe local viscoelastic properties from small sample volumes over a wide frequency range^[^^28^^-^^31^^]^.

There are two general categories for microrheology: active and passive^[^^32^^]^. In the active method, embedded micro-sized probe particles are manipulated by external forces. In the passive method, the probe particles are driven solely by thermal forces, and the intrinsic Brownian motion of the particles is monitored. The motion contains information about the local surrounding microenvironment^[^^33^^]^. In passive microrheological experiments, the trajectory of the probe particles is measured, and the mechanical properties of the surrounding medium are determined through the well-known generalized Stokes-Einstein relation^[^^34^^]^. 

In our previous work, microrheological responses of RADA16-I hydrogel were analyzed using the RSM^[^^35^^]^. Employing the RSM minimizes the number of experimental sets required to model and optimize the intended responses, such as viscoelastic properties^[^^36^^-^^38^^]^. In this study, we examine the impact of experimentally controllable parameters (peptide concentration, added NaCl salt concentration, and milieu type) on the mechanical properties of SPG-178 hydrogels through passive microrheology and RSM. For this purpose, various experiments were performed according to the sets proposed by Design-Expert Software version 7.0. In addition, the optimum values of the experimentally controllable parameters were determined to achieve the highest stiffness for the SPG-178 hydrogels. To the best of our knowledge, no report has been published in the literature on microrheological studies of the SPG-178 hydrogel and optimization of its viscoelastic properties using RSM.

## MATERIALS AND METHODS

**Materials**

SPG-178 peptide, as lyophilized powder, was purchased from ShineGene Molecular Biotech (China). Human blood serum specimens were prepared from a single reference and filtered using a 0.2-micron syringe filter. NaCl salt was procured from Merck (USA). Polystyrene particles (78452, diameter 2 ± 0.05 µm) were provided as a 10 wt% aqueous suspension by the manufacturer (Sigma-Aldrich, USA).

**Sample preparation and experimental procedures**

Samples were prepared by mixing the desired proportions of colloidal particles, peptide solutions, salt solutions, and milieu. Aqueous stock solutions of the particles were prepared at the concentration of 150 ppm by diluting with DI water. Aqueous stock solutions of peptide were prepared at the concentrations of 1.5, 5, 8.5, and 10 g/L by dissolving appropriate amounts of SPG-178 in DI water, followed by sonication for 30 min. In addition, aqueous stock solutions of NaCl were prepared at the concentrations of 120, 410, 700, and 820 mM by dissolving appropriate amounts of NaCl in DI water. Due to the importance of blood-material interaction in clinical applications of SApeptides, half of the tests were carried out in the serum milieu. A leak-proof oval-shaped cavity channel with a diameter of ~1.5 cm was cut into parafilm and attached on a glass microscope slide. Then 10 μL of the desired peptide, salt, and milieu solutions and 3 μL of the colloidal suspension were poured into the cavity and mixed by pipetting to prepare the gel compositions. Afterwards, the loaded cavity was covered with a 0.17 mm-thick glass coverslip. It should be noted that a central composite design was employed for experiments in RSM to investigate the effects of the experimentally controllable parameters. The parameters were coded at the levels of -1.4, -1, 0, +1, and +1.4 with the peptide concentration being set in the range of 0-3 g/L, NaCl concentration in the range of 0-246 mM, and the milieu type being either DI water or the serum (Table 1). Twenty-four sets were suggested by Design-Expert Software (version 7.0) for passive microrheology experiments (Table 2).

**Table 1:** Coded levels of experimentally controllable parameters for experiment design

**Variables**	**Ranges and levels**				
	**-α (-1.4) **	**-** **1 **	**0**	**+1**	**+α (1.4)**
Peptide concentration (g/L)	0	0.45	1.5	2.55	3
NaCl concentration (mM)	0	36	123	210	246
Milieu type	DI water or serum				

Passive microrheology experiments were conducted with an inverted optical microscope equipped with a 60×/NA 1.0 water immersion objective lens (UMPLFLN 60XW, Olympus, Germany) by monitoring the trajectories of the colloidal particles using a CCD camera (at the rate of 30 fps). In a typical experiment, a movie with duration of 10 s was recorded from a probe particle, and the movie was converted to 300 frames. The trajectory of the particle was then determined through the recorded movie. Afterwards, the formalism explained by Dasgupta *et al.*^[^^39^^]^ was used to calculate viscoelastic properties from colloidal inclusion thermal fluctuations. In this vein, after recoding the time series, the MSD was calculated. Then a local power-law was assigned to the MSD. The power law was determined by the logarithmic time derivative of the MSD. In this method, a second-order polynomial fitting with a Gaussian window was used to calculate the first and second logarithmic time derivatives of the MSD. Finally, the generalized Stokes-Einstein relation was employed to calculate the storage $G^{'}(\omega )$ and loss $G^{\mathrm{''}}(\omega )$ moduli as the functions of angular frequency ω.

**Table 2:** Measured and predicted Gʹ and tan δ, with equations 1-4, for experimental sets

**Run number**	**Peptide ** **concentration (g/L)**	**NaCl** **concentration** **(mM)**	**Milieu** **type**	**Measured Gʹ (Pa) ** **at 10 rad s** ^-1^		**Measured tan δ** **at 10 rad s** ^-1^	**Predicted Gʹ (Pa) ** **at 10 rad s** ^-1^	**Predicted tan δ** **at 10 rad s** ^-1^
1	0.45	36.00	DI water		0.30	4.17	0.06	5.51
2	2.55	36.00	DI water		0.35	2.50	0.73	1.73
3	0.45	210.00	DI water		3.56	0.94	1.09	1.42
4	2.55	210.00	DI water		2.96	0.60	4.54	0.63
5	0.00	123.00	DI water		0.005	11.6	0.23	3.78
6	3.00	123.00	DI water		5.78	0.78	2.87	0.88
7	1.50	0.00	DI water		0.067	2.66	0.12	3.5
8	1.50	246.00	DI water		2.18	0.92	3.13	0.72
9	1.50	123.00	DI water		2.87	0.82	3.24	0.79
10	1.50	123.00	DI water		3.10	0.60	3.24	0.79
11	1.50	123.00	DI water		3.36	1.00	3.24	0.79
12	1.50	123.00	DI water		4.20	0.85	3.24	0.79
13	0.45	36.00	Serum		3.50	0.47	1.34	0.65
14	2.55	36.00	Serum		3.55	0.86	4.95	1.06
15	0.45	210.00	Serum		3.37	1.00	1.39	1.68
16	2.55	210.00	Serum		7.08	0.59	6.80	0.58
17	0.00	123.00	Serum		0.012	9.86	1.13	1.27
18	3.00	123.00	Serum		9.20	0.85	7.75	0.85
19	1.50	0.00	Serum		2.68	1.00	2.24	0.68
20	1.50	246.00	Serum		2.08	0.79	3.35	0.72
21	1.50	123.00	Serum		4.95	0.97	4.17	0.86
22	1.50	123.00	Serum		2.32	0.67	4.17	0.86
23	1.50	123.00	Serum		3.74	0.93	4.17	0.86
24	1.50	123.00	Serum		4.90	0.98	4.17	0.86

## RESULTS

**Fig. 1:**
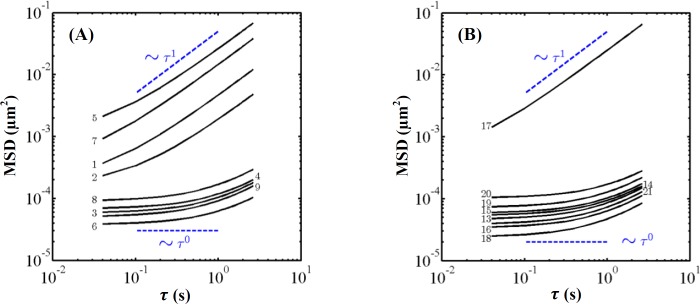
MSD of probed particles versus lag time τ in DI water- (A) and the serum-based (B) experiments. The numbers indicate the corresponding sample number shown in Table 2. The dashed lines are presented merely to guide the eye of diffusive and sub-diffusive motions

**Fig. 2:**
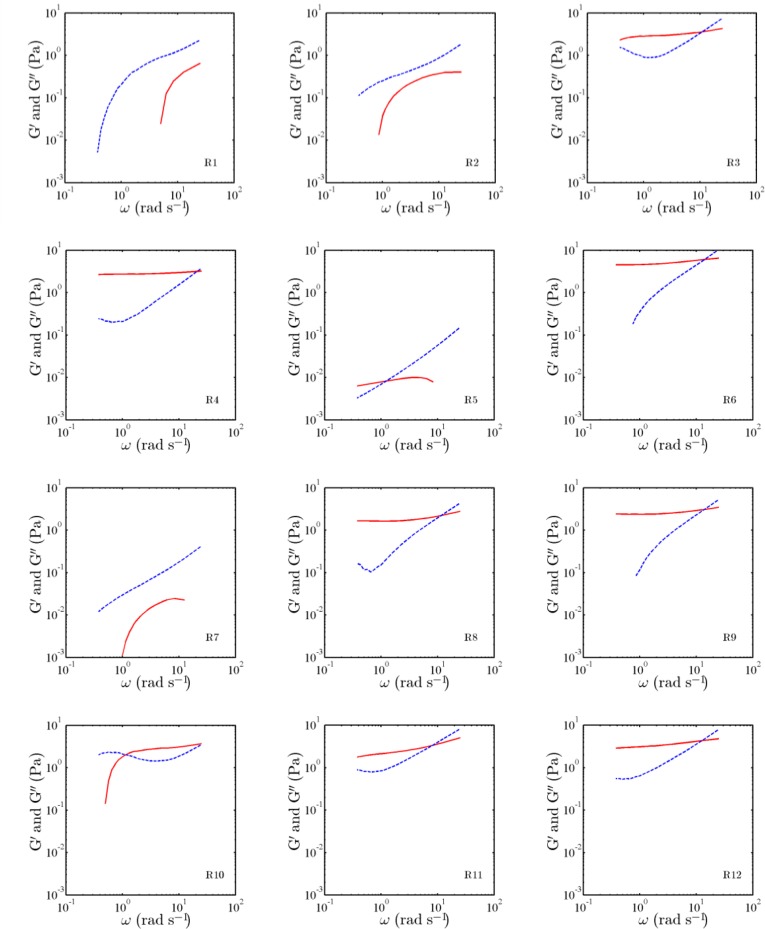
Storage (solid) and loss (dashed) moduli, as a function of angular frequency ω, for the experimental sets R1-R12 listed in Table 2.

**Fig. 3:**
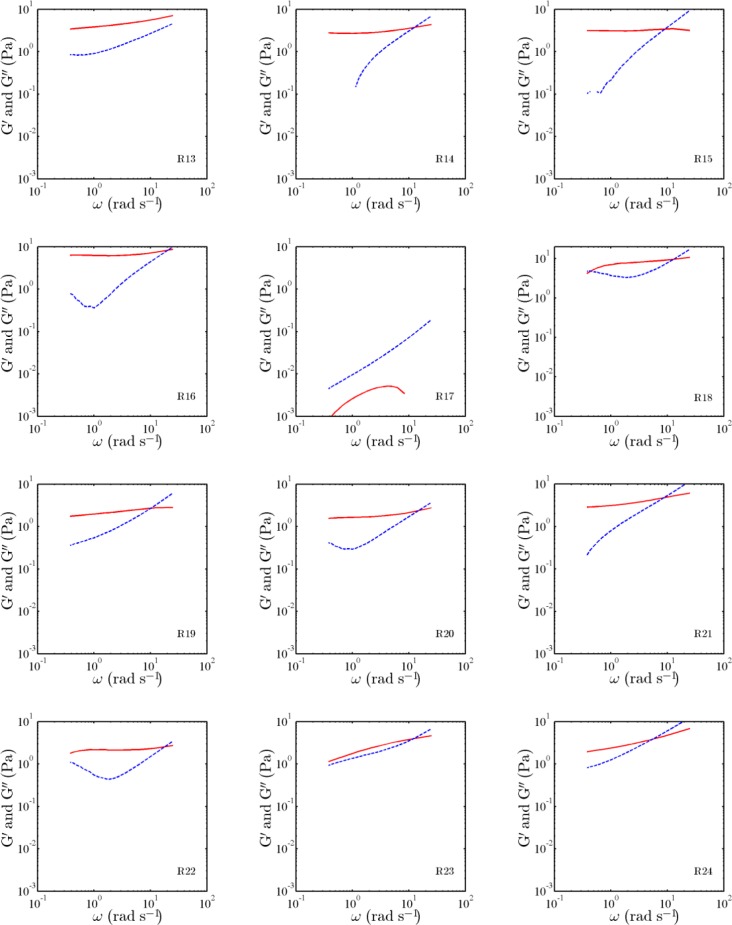
Storage (solid) and loss (dashed) moduli, as a function of angular frequency ω, for the experimental sets R13-R24 listed in Table 2

The measured MSDs of the probed particles embedded in the experimental sets of Table 2 are illustrated in Figure 1, as a function of lag time τ. Also, the measured storage modulus and tan δ of the experimental sets are shown in Table 2, at the frequency of 10 rad s^-1^. In addition, the moduli for the sets, as a function of the angular frequency, are presented in Figures 2 and 3.

ANOVA procedures were performed, and a quadratic polynomial model was fitted to the

experimental data for the storage modulus at the frequency of 10 rad s^-1^. The best model equations for both milieus, which consider main influences (SPG-178 peptide concentration, NaCl concentration, and milieu type), two-factor interactions, and curvature influences, are as follows:

G'0.24=-0.0249+0.636CSPG-178+8.898×10-3CNaCl+1.255×10-5CSPG-178CNaCl-0.147CSPG-1782-2.440×10-5CNaCl2 for DI water 

(1)

G'0.73=0.462+0.931CSPG-178+0.0110CNaCl+2.160×10-3CSPG-178CNaCl-0.0252CSPG-1782-4.779×10-5CNaCl2 for the serum,

(2)

where $C_{\mathrm{SPG}-178}$ and $C_{\mathrm{NaCl}}$ are SPG-178 peptide and NaCl concentrations, respectively. R values of the fitted equations were 0.91 (for DI water) and 0.84 (for the serum). The calculated values of the storage modulus, from the polynomial models, are shown in Table 2, demonstrating that the predicted values from the models are reasonably comparable with the experimental ones.

Another experimental quantity relevant to the mechanical characteristics of hydrogels is tan δ. It represents the ratio of $G^{\mathrm{''}}$ and $G^{'}$. In this study, tan δ was calculated by taking the moduli values at the frequency of 10 rad s^-1^. ANOVA procedures were performed, and a quadratic polynomial model was fitted to the experimental data for the tan δ. The best model equations for both milieus are as follows:

tanδ)-0.72=-0.271+0.746CSPG-178+8.147×10-3CNaCl+6.495×10-4CSPG-178CNaCl-0.196CSPG-1782-2.295×10-5CNaCl2 for DI water 

(3)

((tanδ)-1.13=+2.122-0.252CSPG-178-0.0136CNaCl+5.356×10-3CSPG-178CNaCl-0.0866CSPG-1782+2.111×10-5CNaCl2 for the serum,

(4)

where R values of the fitted equations were 0.92 (for DI water) and 0.69 (for the serum). The calculated values of the tan δ, from the polynomial models, are shown in Table 2, demonstrating that the predicted values from the models are reasonably comparable with the experimental ones.

**Fig. 4:**
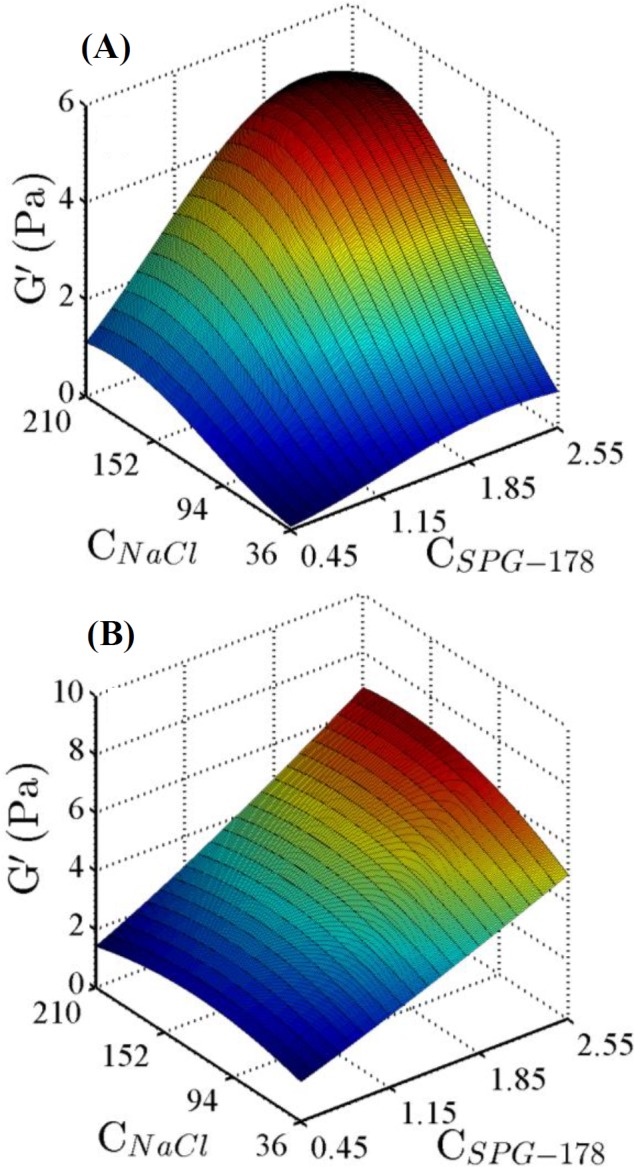
Effects of the SPG-178 peptide and NaCl concentrations on the storage modulus of the SPG-178 hydrogels, predicted by equations 1 and 2, in the milieus of DI water (A) and serum (B)

The surface responses of the storage modulus are presented in Figure 4, with the aid of the statistical models developed for the storage modulus as a function of the experimentally controllable parameters. Furthermore, the values of the experimentally controllable parameters were optimized to achieve the highest value of $G^{'}$and the lowest value of tan δ, exploiting the numerical optimization method provided by Design-Expert 7.0 Software. For the optimization, the SPG-178 concentration was set between 0.45 and 3 g/L. The optimum value for the SPG-178 concentration was obtained in 3 g/L. The milieu type had to be the blood serum to achieve the optimization objective. Also, the optimum value for NaCl concentration was different in the DI water and blood serum.

## DISCUSSION

In the MSDs, two distinct regimes are observed as depicted in Figure 1. In the first regime, the MSDs scale as $\sim \tau^{1}$, indicating diffusive motion in a viscous fluid. In the second one, the MSDs scale as $\sim \tau^{0}$, indicating sub-diffusive motion in a viscoelastic medium^[^^31^^]^. The diffusive motion occurs within samples 1, 2, 5, 7, and 17, where the sol-gel transition is not observed; however, the sub-diffusive motion occurs within the other samples, indicating the sol-gel transition for the corresponding samples.

The characteristics of the measured storage and loss moduli, reported in Figures 2 and 3, are in agreement with the characteristics of probed motions observed in Figure 1. For samples 1, 2, 5, 7, and 17, the storage modulus does not have a distinct low-frequency plateau. In the samples, at low frequencies, the storage modulus is less than the corresponding loss modulus. On the other hand, for other samples, the storage modulus has a distinct low-frequency elastic plateau, and a cross-over frequency is evident. For such samples, the storage moduli are relatively high compared to those for samples 1, 2, 5, 7, and 17.

According to Figure 4, both the SPG-178 peptide and NaCl concentrations modulate the surface responses. Interestingly, in the surface shown in Figure 4B, there is plateau in relation to NaCl concentration, indicating that variation of the NaCl concentration does not considerably affect the surface response in the serum. However, NaCl concentration considerably modulates the surface response in DI water, and increasing NaCl concentration results in the increment of the response.

With the elevation of NaCl concentration, the peptides self-assemble once the electrostatic repulsions among the peptides become less than hydrophobic attractions. For each peptide, there is a certain added-salt concentration, so-called CCC, where at concentrations beyond the CCC, the corresponding peptide becomes a gel^[^^40^^]^. At such concentrations, repulsive forces among peptides vanish, the peptides aggregate, and the corresponding environment becomes a gel.

To understand how the added salt modulates the interactions among the SPG-178 peptides, we considered the structure of the SPG-178 peptide. The peptide has 7 hydrophobic (6 leucine and 1 alanine) and 6 hydrophilic (4 arginine and 2 aspartic acid) residues. Leucine has a large hydrophobic side chain^[^^41^^]^. Thus, with a slight NaCl induction, hydrophobic forces become dominant over repulsive forces and the peptides rapidly converge to cylindrical β-sheet structures, in which the hydrophobic sides are inserted into the interior of the fiber^[^^11^^]^.

It must be recalled that the effect of NaCl concentration on the storage modulus in DI water is more than that of the serum. There are various cations and anions in the serum that affect the self-assembly, even in the absence of NaCl. Thus, in the absence of NaCl, while the electrostatic repulsions are considerable in DI water, the interactions are screened in the serum. Accordingly, the addition of NaCl is of importance in DI water but not in the serum. Further research on the effect of the serum content on the viscoelastic properties of the corresponding hydrogels is recommended.

To sum up, we combined microrheology and RSM for comparative and comprehensive analysis of expensive polymeric drugs and gels. By integrating microrheology and RSM, the viscoelastic properties of SPG-178 peptide hydrogels were examined considering the impact of the SPG-178 peptide and NaCl concentrations, and milieu type. Statistical models were obtained, accounting for the impact of the experimentally controllable parameters on the viscoelastic properties. The statistical models were employed for finding the optimum value of the parameters to maximize the storage modulus and minimize tan δ. The optimum self-assembling condition for SPG-178 SApeptides was in the blood serum with the peptide concentration of 3 g/L. In conclusion, upon the type of materials and available facilities, other microrheological techniques, such as multiple particle tracking microrheology, optical tweezers, and microfluidics could be integrated with statistical methods to optimize soft matter formulations for a variety of applications.

## CONFLICT OF INTEREST.

None declared.
